# Modelling the Nexus of municipal solid waste sector for climate resilience and adaptation to nature-based solutions: A case study of Pakistan

**DOI:** 10.1016/j.heliyon.2024.e31235

**Published:** 2024-05-21

**Authors:** Asif Iqbal, Abdullah Yasar, Abdul-Sattar Nizami, Imran Ali Sultan, Rafia Haider, Amtul Bari Tabinda, Aman Anwer Kedwii, Muhammad Murtaza Chaudhary, Muhammad Usman Ghori

**Affiliations:** a Sustainable Development Study Center (SDSC), Government College University, Lahore 54000, Pakistan; b Civil Services Academy, Government of Pakistan, Lahore 54000, Pakistan; c Deputy Commissioner Office, Government of the Punjab, Lahore 54000, Pakistan; d The Urban Unit, Planning & Development Department, Government of the Punjab, Lahore 54000, Pakistan; e Department of Pharmacy, School of Applied Sciences, University of Huddersfield, Huddersfield HD1 3DH, UK; f Department of Chemical Sciences, School of Applied Sciences, University of Huddersfield, Huddersfield HD1 3DH, UK

**Keywords:** Climate resilience, Waste & ecosystems, Waste management, Ecological impact value (WS-EIV), Nature-based solutions

## Abstract

Municipal solid waste management is a major concern in developing economies, requiring collective international efforts to achieve carbon neutrality by diverting waste from disposal facilities. This study aims to highlight the importance of the waste sector as it has the potential to significantly contribute to climate change and its toxicity impact on the local ecosystem. Out of the total municipal solid waste generated, only 78 % is collected, either open dumped or thrown in sanitary landfills. The waste sector's ecological impact value is calculated for the Earth's regions, and it is very high at >50 % in Africa, Asia, Latin America and the Caribbean. This sectoral impact value is mainly responsible for greenhouse gas emissions and degradation of the local ecosystem health. Current business‒as‒usual practices attribute 3.42 % of global emissions to the waste sector. Various scenarios are developed based on waste diversion and related emissions modelling, and it is found that scenarios 3 and 4 will support the policymakers of the regions in attaining zero carbon footprints in the waste sector. Our findings conclude that cost-effective nature-based solutions will help low‒income countries reduce emissions from disposal sites and significantly improve the local ecosystem's health. Developed economies have established robust waste‒handling policies and implementation frameworks, and there is a need for collaboration and knowledge sharing with developing economies at the regional level to sustain the sector globally.

## Highlights

• A solid waste management model was proposed to achieve zero carbon footprints.
• Climate impact of waste sector is analyzed for global temperature and related sea level rise.
• Waste sector is linked with ecosystem health using a novel methodology.
• A matrix is developed to assess the hazard impact level of the waste sector.
• Cost-effective nature-based solutions (NbS) and adaptation approaches are focused.

## Graphical abstract

**Figure:**
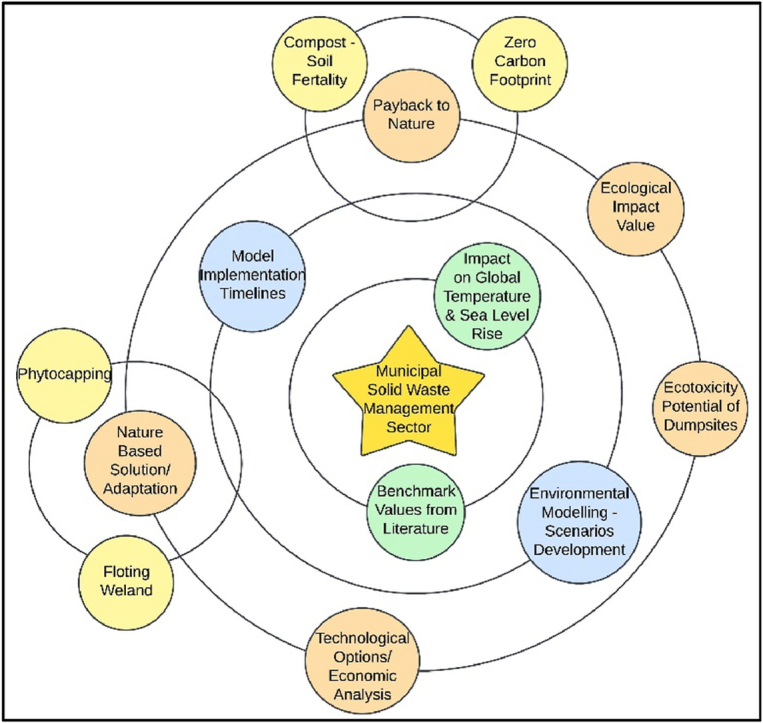

## 1 Introduction

Municipal Solid Waste (MSW) management is a critical concern for developing economies to sustain the living standard in urban areas, and efforts are more focused on its collection to clean the vicinity and dump waste in the periphery of the city without considering its adverse impacts on local ecosystem, environment and global climate [1]. Most developing economies around the globe are in a transition phase to attain sustainability in the waste sector [2]; however, the economic conditions, technological barriers, and local capacity issues hinder achieving the desired results. The waste generation quantum is projected to grow with the increase in population and urbanization with uncertainty in waste diversion for treatment [3]; however, the waste generation rate is low in developing economies compared to developed economies as it is linked with the area's economic condition [4]. Ecosystem health and biodiversity are adversely affected by the expansion of urban land use and related increases in pollution levels due to anthropogenic activities [5]. The waste sector, in terms of infrastructure such as storage, treatment and disposal sites, has the ecotoxicity potential to degrade the local ecology as these storage sites are unevenly distributed in urban areas, and disposal facilities are considered hotspots for surrounding environment with climate change impacts which primarily located in remote regions across various geographic scale [6].

European Union (EU) has developed policies to achieve the targets for waste diversion from landfills, which helps sustain the sector environmentally under green technological interventions, resulting in reduced greenhouse gas (GHG) emissions [7]. However, in Germany, Denmark, Belgium, Netherland, and the United Kingdom (UK), the MSW disposal share in landfilling is <20 % compared to other European countries such as Bulgaria, Greece, Croatia, and Iceland, where >60 % of MSW is dumped in landfills [8]. United States of America (USA) has installed more than 86 waste-to-energy (WtE) facilities based on refuse-derived fuel (RDF) and mass burn technologies and committed to diverting the waste from landfilling under various technological advance pilot projects for gasification and pyrolysis to minimize the exhaust of toxic substances in the air to safeguard the local environment and ecosystem [9]. Variation in waste disposal tonnage is observed in the USA. A study analysis shows 115 % more waste disposal in landfills than Environmental Protection Agency (EPA) estimates, with at least one fire incident reported at 46 % of landfill facilities [10]. China is in a transitional phase to integrate the reduce, reuse and recycle strategy in waste handling, focusing on behavioural changes towards food waste minimization to reduce related carbon footprint [11].

China also implemented a new waste separation strategy in 2017 that helped the country improve the recycling rate by >33 % with increased waste management efficiency. This intervention has positively impacted citizen satisfaction levels with an overall reduction in GHG emissions [12]. It is suggested that a material recovery facility (10.13039/501100000403MRF), compost manufacturing from kitchen waste and incineration, including WtE, are more feasible solutions for Dammam, Kingdom of Saudi Arabia's waste handling, and these interventions will support the policy formation on clean energy production with a reduction in GHG emissions [13].

In developing economies, city-level emissions from the waste sector are calculated as 1.4 to 2.6 times more than the actual reported values in emissions inventories and disposal sites are found to contribute up to 50 % of those emissions [14]. Therefore, the waste management sector can contribute to safeguarding the local environment and ecosystem by increasing the waste recovery rate for organic compost and RDF as per the composition and quality of MSW in developing economies [15]. A shift of the current waste paradigm from landfilling and open disposal to anaerobic digestion, mechanical biological treatment, incineration, WtE and landfill gas (LFG) recovery with sophisticated technologies can support the municipalities to attain environmental sustainability. The LFG generation and its collection efficiency are affected by the quantity and quality of the tonnage dumped, waste decay rate, and the presence of non‒methane organic compounds, which makes landfills a more suitable choice as compared to incineration from the perspective of GHG emissions [16].

Waste diversion for recycling can support municipalities in reducing carbon emissions by 0.21 % and increase economic growth by 0.32 % against each 1 % increase in recycling rate [17]. However, there is a need to incorporate climate action and smart city approaches into local policies for integrated waste management systems [18]. Integrating dedicated separate waste collection streams, maximum recovery of recyclables, and establishment of WtE projects with minimal waste for landfilling will help the MSW sector emerge as a carbon sink globally [7]. Open disposal of waste is a common practice in developing countries due to the availability of land depression around the cities [19]. The waste is used as material to fill the Earth's depression and reclaim the land for further utilization by the land owner; however, this practice is an environmental hazard responsible for 14 % of estimated global GHG emissions with third prominent source of anthropogenic methane [20] and potential health risks for adjacent populations and local ecology due to soil contamination with heavy metals [21].

This waste mishandling negatively impacts public health, ecosystems, and biodiversity, contaminating ground and surface water, soil, and the atmosphere. The decomposition of organic waste produces leachate that can lead to aquatic pollution if disposed of near the water body. In that case, there is a strong possibility of such incidents in developing countries, and such practices may lead towards a lethal impact on ecosystem health [22]. Areas along waste disposal sites are considered vulnerable ecological risk regions having low self-equilibrium ability. Therefore, there is a need for scientific handling and diversion of MSW from landfilling with proper soil remedial measures plans to sustain the local ecosystem's health [23]. Nature-based solutions (NbS) are gaining social acceptance as green technology [24], such as the application of phytocapping techniques to rehabilitate old waste disposal sites with native plant species, can potentially reduce the leachability of heavy metals into groundwater [25], act as hyper-accumulator for high-risk containments [26] and also reduce surface methane emissions into the atmosphere by 45 % compared to un-vegetated sites [27].

The NbS has the great potential to transform the urban hotspot into green infrastructure with value-added public assets by integrating regenerated spaces with city infrastructure [28]. Various international agreements and agendas, i.e., Paris Agreement, sustainable development goals (SDGs), New Urban Agenda (NUA), and decade of ecosystem restoration, focus on sustainable development, climate change mitigation and its integration, and protecting and restoring the ecosystem. Further, Nationally Determined Contributors (NDCs) and green fence campaigns [29], have restrictions and quality control checks on the transboundary movement of coal and plastic waste, respectively, which resultantly created an opportunity to focus on green technological interventions to explore the utilization of high caloric waste as a conventional fuel substitute to strengthen the local energy sector. Smart city industry can initiate economic growth with high emissions costs that require product modification at the manufacturing level for emissions reduction [30].

The United Nations emphasized collective efforts towards a global action plan in reducing GHG emissions to limit global warming to well below 2 °C by putting robust policies for member nations to achieve emissions reduction targets; however, it seems that the world may overshoot the global emission reduction budget [31]. The share of the waste sector in global GHG emissions may account for 5 % [32]. There is an urgent need to address the gaps in waste treatment technology, related infrastructure, financial resources, and legislation that contribute to the accumulation of waste at disposal sites in developing economies. Technology transfer with financial investment can help reduce global inequality in waste and promote the climate resilience approach [33]. This waste‒related anthropogenic activity is responsible for transitioning from the geological Holocene towards the Anthropocene epoch [34].

**Fig. 1:**
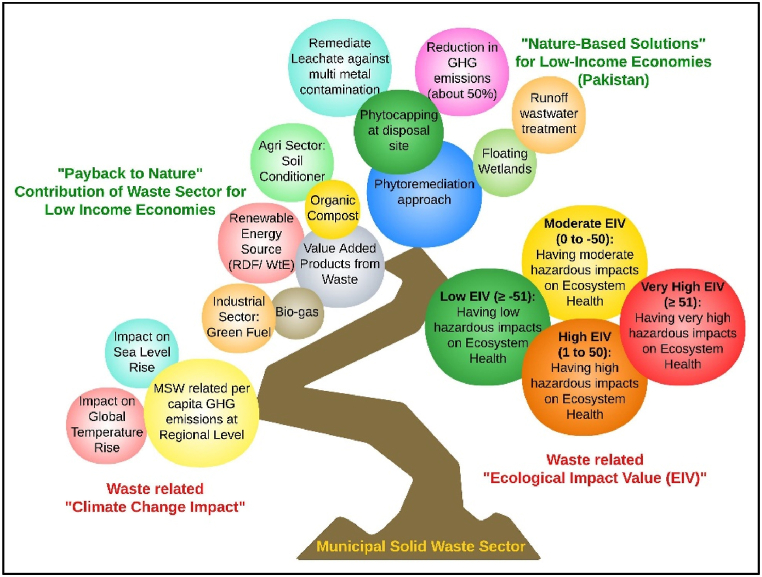
Theme and novelty of the study/research work.

Henceforth, this study aims to understand and examine the impact of waste on climate change and the local ecosystem. The novel work is performed to assess the waste sector's related ecological impact value (WS-EIV) and related share of waste sector in global temperature and sea level rise, as depicted in Fig. 1. The sustainability of the waste sector from the perspective of various treatment technologies, operational costs and related net climate impacts is highlighted for policy decisions. Based on our findings, we shall discuss and make future recommendations for the sustainability of the most at‒risk regions from the perspective of waste handling. Our results are expected to inform policymakers and international lenders to initiate efforts in transforming environmental legislation and policies with nature-based, cost-effective solutions in national and regional waste strategies by incorporating multi-scalar approaches to mitigate the waste-related climatic and ecological impacts to safeguard the environment for developing economies. The potential of waste sector to pay back nature is also highlighted, as explained in Fig. 1.

Furthermore, to highlight the MSW-related carbon neutrality opportunities at the regional level in perspective of climate change impacts, green technological intervention in combination with NbS for a better policy decision by regional and international bodies for sustainable development [35] such as International Institute for Sustainable Development (IISD), Asian Think Tanks Network (ADB-ATTN), Central European Initiative (CEI), Intergovernmental Authority of Development (IGAD), The Pacific Community (SPC), The Union of South American Nations (Unasur), Forum of East Asia and Latin American Corporation (FEALAC), The Caribbean Community (CARICOM), Organization of American States (OAS), Shanghai Corporation Organization (SCO), Israel Public Policy Institute (IPPI), South Asian Association for Regional Corporation (SAARC), Indian Institute of Sustainable Development (IISD), Council on Energy Environment and Water (CEEW), UN Habitat, Korean International Urban Training Center (IUTC), UN Environment Programme Copenhagen Climate Center (UNEPCCC), Global Network of National Councils for Sustainable Development and Similar Bodies (GN-NCSDs), German Council for Sustainable Development (RNE), Sustainability First, Governance and Policy Think Tank (GPTT), Ideation Center, Sustainable Development Policy Institute (SDPI), and Institute of Strategic Studies Islamabad (ISSI).

## 2 Materials and methods

### 2.1 Research framework and area

The research framework is developed to analyze the global environmental emissions from the MSW sector. A holistic approach is applied to create scenarios (S) to further develop the model from the perspective of waste diversion targets from landfilling to convert the diverted waste into value-added green products. These waste diversion targets are defined at 25 % (S-1), 50 % (S-2), 75 % (S-3) and 85 % (S-4) to reduce the waste disposal burden in landfilling, and this approach will support GHG emissions reduction in a phased manner to achieve an overall carbon neutrality of waste sector. The policy documents on municipal solid waste handling in developed and developing economies support this rationale for waste diversion from landfilling as defined in scenarios development. Implementing the proposed emission reduction model for the Earth regions will help towards targeted goals of waste diversion to safeguard the global climate and ecosystem health.

**Fig. 2:**
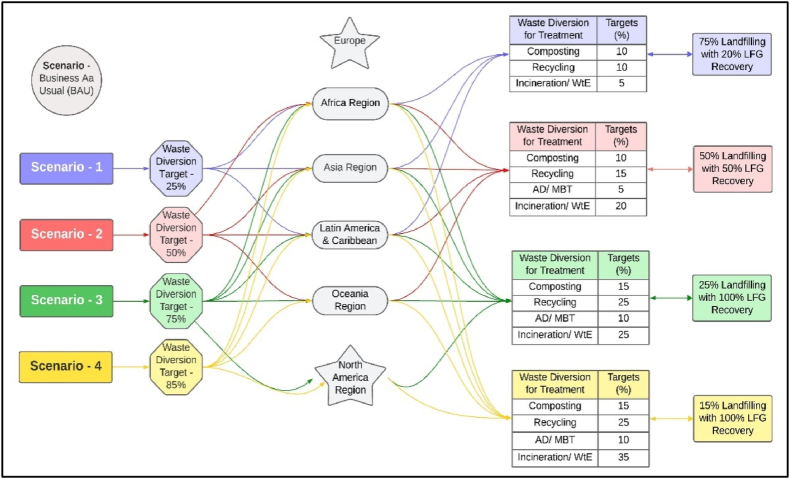
Research formwork, areas and assumptions used for scenario development to perform environmental modelling.

Furthermore, the areas are selected based on the Earth's land divisions, i.e., Africa, Asia, Europe, Latin America and the Caribbean (LAC), North America (NA), and Oceania. All the regions are included in business‒as‒usual (BAU) scenario; however, Europe is excluded from further scenarios as this region has already achieved the proposed waste diversion collective target (this region only needs to focus on technological interventions to reduce emissions of black carbon), the NA region is excluded from S-1, and S-2, and Oceania region is excluded from S-1 based on waste diversion targets from landfilling. Details of the research framework, areas and assumptions used to develop various scenario models are depicted in Fig. 2. A case study of Pakistan's waste sector is also performed to highlight the areas for improvement.

### 2.2 Data Mining and benchmark values

The secondary data (Table 1) is accessed from the existing literature on world population, physical composition of MSW, current waste treatment and disposal trends, GHG emissions share in global warming, i.e., global temperature rise (GTR) and sea level rise (SLR). Secondary data helps define the benchmark values for further analysis, modelling and scenarios development.

**Table 1:** Secondary data accessed from literature as benchmark values.

Material/literature for waste modelling							References
Population data of the geographical regions							[36]
Assessment of current waste generation rate							[32]
Per capita GHG emissions from all sources and sectors							[37]
Data on global warming, i.e., GTR and related SLR from 1990 to 2100							[38,39]
The region-wise current MSW physical composition is used as benchmark in scenarios development;							
Components	Africa	Asia	Europe	LAC	NA	Oceania	[4,7,9,18,40,41]
Organic (%)	57	47.7	35	54	35	51	
Plastic (%)	13	9.9	10	12	13	17	
Paper (%)	9	16.6	25	16	26	4.1	
Glass (%)	4	3.8	8	4	4	4.1	
Metal (%)	4	3.1	5	2	11	6.6	
Textile (%)	0	3.5	5	0	9	3.8	
Other (%)	13	15.4	12	12	2	13.4	
The regional trends of current waste treatment and disposal methods that are used as benchmark in scenarios development;							
Treatment/Disposal Methods	Africa	Asia	Europe	LAC	NA	Oceania	[8,10,18,20,31,32,[42], [43], [44], [45], [46]]
Recycling (%)	4	9.2	48	4.5	11.2	9	
Open dumping (%)	47	45.1	–	33	–	19	
Composting (%)	0	1.5	–	10	27	2	
Landfill (%)	29	19.8	23	51.5	50	46	
Incineration/WtE (%)	2	6.6	29	1	12	24	
Others, including open burning (%)	18	17.8	–	–	–	–	
Landfill gas (LFG) recovery (%)	1	3.5	35	9	42	14	

### 2.3 GHG emissions Calculation – environmental model

Waste sector-related GHG emissions are calculated by applying an emission quantification tool (EQT) for the estimation of GHG/short-lived climate pollutants (SLCP) from the waste sector based on country-specific emissions factors and Intergovernmental Panel on Climate Change (IPCC) recommended default values as developed by the Institute for Global Environmental Strategies (IGES), Japan [47], which follows the lifecycle assessment (LCA) approach to account for actual and projected waste-related emissions, and special care was ensured regarding assumptions on the dominant climate prevailing in the regions. Furthermore, the following assumptions were implied in scenarios, including 100 % utilization of compost for agriculture purposes, utilization of heat and biogas as a substitute for coal and natural gas, recovery of compost from anaerobic digestion (AD), use of compost material byproduct of mechanical biological treatment (MBT) process, as fertilizer to substitute chemical fertilizer, continuous stoker incineration type, operational activities based on diesel consumption, sanitary landfilling (25 years life) with landfill gas recovery (LFG) with electricity and heat production.

**Fig. 3:**
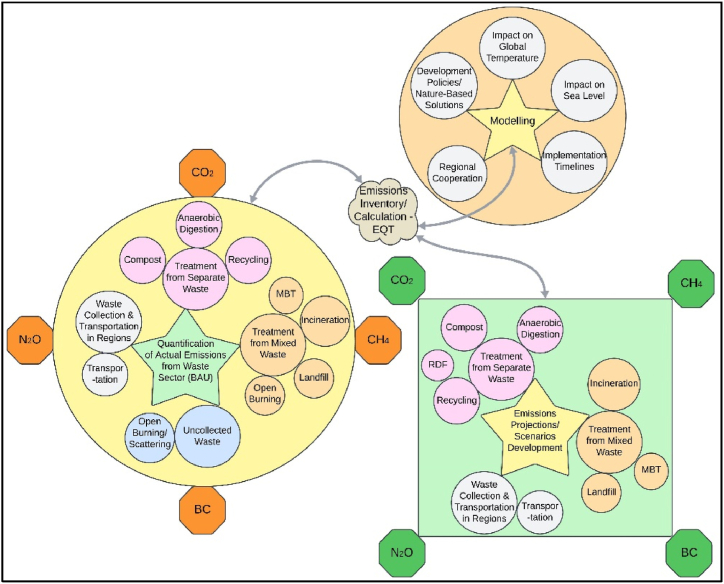
Scope of work of the study.

The holistic approach is applied to assess the emissions for the waste sector, scenarios development, and related modelling for emissions projections based on waste diversion proportion using EQT, which has inventory data of countries specific for recycling and other waste treatment options [47]. Primary input data, such as the quantity of waste, its composition, fossil fuel consumption for waste collection/fleet operations [48], grid electricity requirement, waste treatment, and recoverable amounts, was inserted in the GHG calculator. The waste treatment methodology, related emissions, gas quantification/projections, modelling, scenarios development and scope of the study are explained in Fig. 3.

### 2.4 Climate change model in perspective of waste sector

The MSW sector emissions share (%) in total GHG emissions (all sectors) is calculated as per following Equation (1);

(1) $${WSE}_{\%Share\left(GHG\right)}=\left({WSE}_{PC\left(GHG\right)}÷{ASE}_{PC\left(GHG\right)}\right)\;X\;100$$

The MSW sector contribution in GTR and related SLR is calculated as per following Equations (2‒6);

(2) $$\left[\begin{array}{ccc} WSS_{GTR\left({}^{\circ}c\right)2024} & & WSS_{SLR\left(cm\right)2024}\\ \begin{array}{c} WSS_{GTR\left({}^{\circ}c\right)2025}\\ WSS_{GTR\left({}^{\circ}c\right)2026}\\ \vdots \end{array} & & \begin{array}{c} WSS_{SLR\left(cm\right)2025}\\ WSS_{SLR\left(cm\right)2026}\\ \vdots \end{array}\\ WSS_{GTR\left({}^{\circ}c\right)2100} & & WSS_{SLR\left(cm\right)2100} \end{array}\right]=\left[\begin{array}{ccc} GTR_{{}^{\circ}c\left(2024\right)}\;X\;{WSE}_{\%Share\left(GHG\right)} & & SLR_{cm\left(2024\right)}\;X\;{WSE}_{\%Share\;\left(GHG\right)}\\ \begin{array}{c} GTR_{{}^{\circ}c\left(2025\right)}X\;WSE_{\%Share\left(GHG\right)}\\ GTR_{{}^{\circ}c\left(2026\right)}\;X\;{WSE}_{\%Share\left(GHG\right)}\\ \vdots \end{array} & & \begin{array}{c} SLR_{cm\left(2025\right)}\;X\;{WSE}_{\%Share\;\left(GHG\right)}\\ SLR_{cm\left(2026\right)}\;X\;{WSE}_{\%Share\;\left(GHG\right)}\\ \vdots \end{array}\\ GTR_{{}^{\circ}c\left(2100\right)}\;X\;WSE_{\%Share\left(GHG\right)} & & SLR_{cm\left(2100\right)}\;X\;{WSE}_{\%Share\left(GHG\right)} \end{array}\right]$$

(3) $$A{AWSS}_{GTR\left({}^{\circ}c\right)}=\left({WSS}_{GTR\left({}^{\circ}c\right)2100}-\;{WSS}_{GTR\left({}^{\circ}c\right)2024}\right)÷Y_{n}$$

(4) $${AAWSS}_{SLR\left(cm\right)}=\left({WSS}_{SLR\left(cm\right)2100}-\;{WSS}_{SLR\left(cm\right)2024}\right)÷Y_{n}$$

(5) $$\left[\begin{array}{ccc} AWSS_{GTR\left({}^{\circ}c\right)2024} & = & AAWSS_{GTR\left({}^{\circ}c\right)}\\ \begin{array}{c} AWSS_{GTR\left({}^{\circ}c\right)2025}\\ AWSS_{GTR\left({}^{\circ}c\right)2026}\\ \vdots \end{array} & \begin{array}{c} =\\ = \end{array} & \begin{array}{c} \sum_{2024∼2025}AAWSS_{GTR\left({}^{\circ}c\right)}\\ \sum_{2024∼2026}AAWSS_{GTR\left({}^{\circ}c\right)}\\ \vdots \end{array}\\ AWSS_{GTR\left({}^{\circ}c\right)2100} & = & \sum_{2024∼2100}AAWSS_{GTR\left({}^{\circ}c\right)} \end{array}\right]$$

(6) $$\left[\begin{array}{ccc} AWSS_{SLR\left(cm\right)2024} & = & AAWSS_{SLR\left(cm\right)}\\ \begin{array}{c} AWSS_{SLR\left(cm\right)2025}\\ AWSS_{SLR\left(cm\right)2026}\\ \vdots \end{array} & \begin{array}{c} =\\ = \end{array} & \begin{array}{c} \sum_{2024∼2025}AAWSS_{SLR\left(cm\right)}\\ \sum_{2024∼2026}AAWSS_{SLR\left(cm\right)}\\ \vdots \end{array}\\ AWSS_{SLR\left(cm\right)2100} & = & \sum_{2024∼2100}AAWSS_{SLR\left(cm\right)} \end{array}\right]$$

### 2.5 Ecological impact model in perspective of waste sector

The waste sector's ecological impact value (WS-EIV) in perspective of service delivery performance, waste collection gap, waste direct disposal, waste diversion for treatment, and LFG recovery, including leachate management values, are calculated for all scenarios, as per the following Equation (7‒12);

(7) $${SDP}_{\%\left(sys\right)}=\left({WC}_{t}÷{WG}_{t}\right)\;X\;100$$

(8) $${WCG}_{\%\left(sys\right)}=100-{SDP}_{\%\left(sys\right)}$$

(9) $${WD}_{\%\left(ttt\right)}={(WD}_{t\left(ttt\right)}÷{WC}_{t})\;X\;100$$

(10) $${WDD}_{\%\left(lfs\right)}=\left({WDD}_{t\left(lfs\right)}÷{WC}_{t}\right)\;X\;100$$

(11) $${LFGR}_{\%}={(WDC}_{t\left(lgr\right)}÷{WDD}_{t\left(lfs\right)})\;X\;100$$

(12) $${WS}_{\%\left(E\pm \right)}={{({WCG}_{\%\left(sys\right)}+WDD}_{\%\left(lfg\right)})-(LFGR}_{\%}+{WD}_{\%\left(ttt\right)})$$

The parameters and variables utilized in Equation (1‒12) are explained in Table 2.

**Table 2:** Parameters and variables for MSW model.

Assessment/calculations for MSW model			
WSE_%share(GHG)_	Waste sector emissions share (in percentage) of GHG	WSE_PC(GHG)_	Per capita GHG emissions from waste sector
ASE_PC(GHG)_	Global per capita GHG emissions from all sources/sectors and value is taken as 6.9 tons from literature [37]	WSS_%GTR(years)_	Annual waste sector share (value in percentage) in global temperature rise
WSS_%SLR(years)_	Annual waste sector share (value in percentage) in sea level rise	GTR_°c(years)_	Annual global temperature rise value is taken from literature [38,39]
SLR_cm(years)_	Annual sea level rise value is taken from literature [38,39]	AAWSS_GTR(°c)_	Average annual share of waste sector in global temperature rise (°C)
AAWSS_SLR(cm)_	Average annual share of waste sector in sea level rise (cm)	AWSS_GTR(°c)_	Actual waste sector share in global temperature rise (°C)
AWSS_GTR(°c)_	Actual waste sector share in sea level rise (cm)	SDP_%(sys)_	Service delivery performance/efficiency in percentage in perspective of MSW collection
WG_t_	Waste generated in tons from the service area	WD_%(ttt)_	Waste diverted (percentage) for treatment, i.e., compost, recycling, AD, MBT, and incineration
WCt	Waste collected in tons by the municipality from the service area	WDD_%(lfs)_	Waste direct disposal (percentage) at landfill site from the collected waste
WD_t(ttt)_	Waste diverted in tons for treatment	LFGR%	Landfill gas recovery in percentage
WDD_t(lfs)_	Waste direct disposal in tons at landfill site	WS_%(E±)_	Waste sector contribution value in percentage in perspective of ecological impact value (WS-EIV)
WDC_t(lgr)_	Waste disposal (tons) in cell method for landfill gas recovery. This method also prevent leachate seepage and runoff	WCG_%(sys)_	Waste collection gap of the system in percentage
Y_n_	Number of years	–	–

### 2.6 Chemical analysis of landfill leachate and organic compost

The leachate and organic compost samples were collected from Sahiwal City to assess its toxic/non-toxic impacts on the local ecosystem and agricultural land. Chemical results of the leachate and organic compost samples were obtained from the laboratory that is authorized by the EPA and the soil and water testing laboratory for research, the Agriculture Department - Government of Punjab, respectively.

## 3 Results and discussion

### 3.1 Waste management practices in the world

**Fig. 4:**
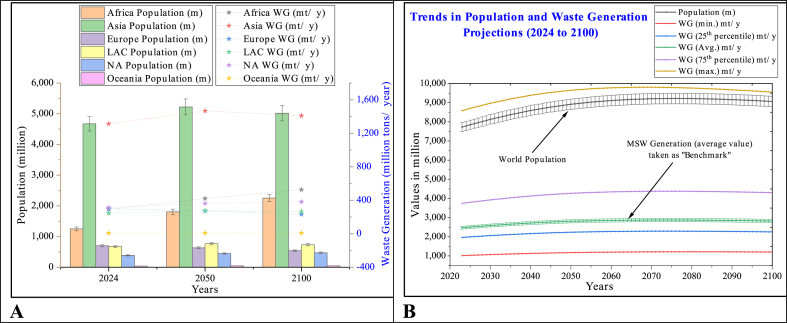
The world population and waste generation trend over the period. (A) Regional population and waste generation. (B) Global population and waste generation projections.

The world's projected population is about 8 billion in 2024 [36]. Asia has the highest population share at 60.4 %, followed by Africa at 16.1 %, Europe at 9.1 %, LAC at 8.7 %, NA at 5 %, and Oceania at 0.5 %. Global waste generation is calculated as 2472 million tons in 2024 based on average waste generation rate [32]. Asia has reported the highest waste generation proportion at 53.1 %, followed by NA at 12.7 %, Europe at 12.2 %, Africa at 11.8 %, LAC at 9.9 %, and Oceania at 0.3 %. NA has reported the highest per capita average daily waste generation rate at 2.21, followed by Europe at 1.18, LAC at 0.99, Asia at 0.77, Africa 0.64, and Oceania at 0.56. The regional population and related waste generation are depicted in Fig. 4a. World population and waste generation quantum are further projected till 2100 (Fig. 4A). Moreover, waste generation quantum projections are based on minimum value, 25^Th^ percentile, average value, 75th percentile and maximum value as depicted in Fig. 2B, however average waste generation value is taken as a benchmark indicator for further scenarios development. The detail of population growth and related MSW generation is depicted in supplementary material.

Currently, the waste collection efficiency is reported at about 80 %, and remaining uncollected 494 million tons (20 %) per annum waste is scattered in vacant places in urban areas, unauthorized disposal in open wastewater drains, and burned in developing economies around the globe. This gap in service delivery is responsible for environmental and biodiversity degradation. Disposing 4.8–12.7 million tons of plastic waste annually into the oceans [49] also impacts marine life and seabirds.

Europe region is on top with highest recycling rate (48 %). LAC and Africa regions have the lowest 4 % and 4.5 % recycling rates, respectively. Oceania region has the highest incineration and WtE rate at 24 % and lowest at 1 % LAC. Open disposal of waste is prominent method in Africa (47 %) and Asia (45.1 %) regions. Highest rate of composting (27 %) is reported in NA region. The highest organic contents (57 %), i.e., kitchen and garden waste, are found in Africa, with the lowest (35 %) in Europe. The waste composition and related treatment and disposal methods are explained in Table 1.

### 3.2 Scenarios development and environmental model for Earth regions

**Fig. 5:**
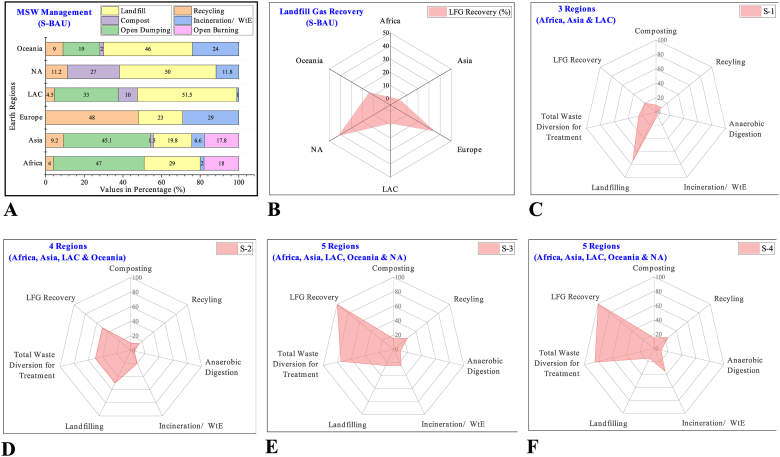
Assumptions used in scenario development. (A) Business as usual Scenario. (B) Landfill gas recovery under business as usual. (C) Scenarion-1. (D) Scenario-2. (E) Scenario-3. (F) Scenario-4.

Five scenarios have been developed to propose and highlight a viable environment-friendly solution towards carbon neutrality from the perspective of the waste sector. The scenario (S-BAU) is based on current benchmark values of waste collection, treatment, and final disposal. Region-wise, MSW management practices and related LFG recovery under S-BAU are depicted in Fig. 5A and B, respectively.

Scenario-1 is developed for three regions due to low waste treatment: Africa at 6 %, Asia at 17.3 %, and LAC at 15.5 %. Therefore, S-1 has proposed a 25 % waste diversion target focusing on recycling and composting technologies. Waste disposal through sanitary landfilling target is defined as 75 % with 20 % LFG recovery as proposed in S-1 (Fig. 5C). Four regions, i.e., Africa, Asia, LAC and Oceania are proposed in S-2 to meet the targets of 50 % waste diversion with more focus on WtE and incineration of waste. The waste disposal target is defined at 50 % through sanitary landfilling with 50 % LFG recovery in S-2 (Fig. 5D). Scenario-3 is proposed to include five regions, i.e., Africa, Asia, LAC, NA and Oceania with 75 % waste diversion target with more focus on recycling and WtE projects. The waste disposal target with LFG recovery is defined at 25 % and 100 % in S-3 (Fig. 5E). S-4 also includes the same regions as in S-3 (see supplementary material). However, the waste diversion target is defined at 85 %, focusing more on WtE and waste incineration as a substitute for conventional fuel for the energy sector. Waste final disposal and LFG recovery targets are defined at 85 % and 100 %, respectively, in S-4, as depicted in Fig. 5F.

**Fig. 6:**
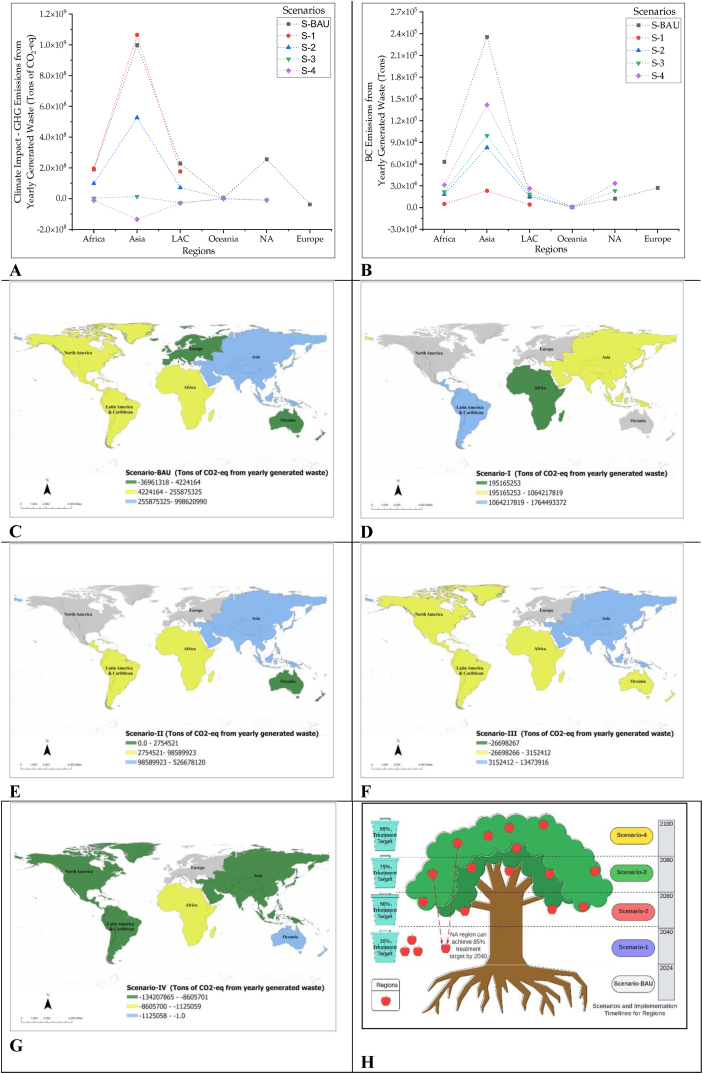
Environmental model for scenarios from the perspective of carbon neutrality. (A) GHG emissions value from each scenario. (B) BC emissions value from each scenario. (C) Geographic representation of emissions value in business as usual. (D) Geographic representation of emissions value in Scenario-1. (E) Geographic representation of emissions value in Scenario-2. (f) Geographic representation of emissions value in Scenario-3. (G) Geographic representation of emissions value in Scenario-4. (H) Implementation timelines for execution of proposed scenarios.

The GHG and SLCP emissions from waste collection, transportation, treatment and final disposal are calculated [47] for each scenario, as depicted in Fig. 6A and B. The yearly collective GHG and black carbon (BC) emissions from all regions under S-BAU are calculated at 1639 million tons CO_2‒_eq and 354,184 tons, respectively. The GHG share of the Asia region is highest, with 998.6 million tons of CO_2_‒eq and 235,315 tons of BC emissions per annum. The regional geographic presentation of GHG emissions in S-BAU is depicted in Fig. 6C. The collective GHG share from three regions in S-1 is calculated as 1436 million tons of CO_2_‒eq and 32,432 tons of BC emissions from yearly generated waste. The regional geographic presentation of GHG emissions in S-1 is depicted in Fig. 6D. The collective GHG share from four regions in S-2 is calculated as 700 million tons of CO_2_‒eq and 116,801 tons of BC emissions from yearly generated waste.

The regional geographic presentation of GHG emissions in S-2 has depicted in Fig. 6E. The collective share of GHG from five regions in S-3 is calculated as −21 million tons of CO_2_‒eq and 163,540 tons of BC emissions from yearly generated waste. The regional geographic presentation of GHG emissions in S-3 is depicted in Fig. 6F. The collective share of GHG from five regions in S-4 is calculated as −184 million tons of CO_2_‒eq and 233,279 tons of BC emissions from yearly generated waste. The regional geographic presentation of GHG emissions in S-4 is depicted in Fig. 6G. Therefore, S-3 and S-4 are environmentally friendly options for the regions regarding GHG emissions reduction targets. However, the higher BC emissions in these scenarios require technological interventions at international level to make waste treatment technologies, especially incineration and WtE, more environment-friendly options.

The waste sector can obtain the carbon neutrality target by implementing the proposed scenarios in phased manner with tentative implementation timelines to achieve environmental sustainability. The objective of proposed scenarios is to provide flexible, practicable implementation timelines for each region to help the sector towards global carbon neutrality by 2080. The European region is excluded from S-1 to S-4 because it has already achieved the collective target of carbon neutrality as explained in S-BAU, however there is only a need to enhance the efficiency of current technologies in perspective of thermal treatment to achieve the neutrality in BC emissions (Fig. 6B). S-1 will help achieve the 12.4 % GHG emission reduction target compared to the benchmark value of S-BAU by 2040. S-2 will help achieve the 57 % GHG emission reduction target compared to the benchmark value of S-BAU by 2060. S-3 and S-4 will help achieve the >100 % GHG emission reduction target compared to benchmark value of S-BAU by 2080 and 2100, respectively.

The NA region has the great potential to achieve carbon neutrality targets by 2040 by implementing the waste diversion targets as explained in S-3 and S-4, and proposed implementation timelines for each scenario are depicted in Fig. 6H. Detail of GHG and BC emissions is explained in supplementary material.

### 3.3 Sensitivity analysis of scenarios and related emissions impacts

**Fig. 7:**
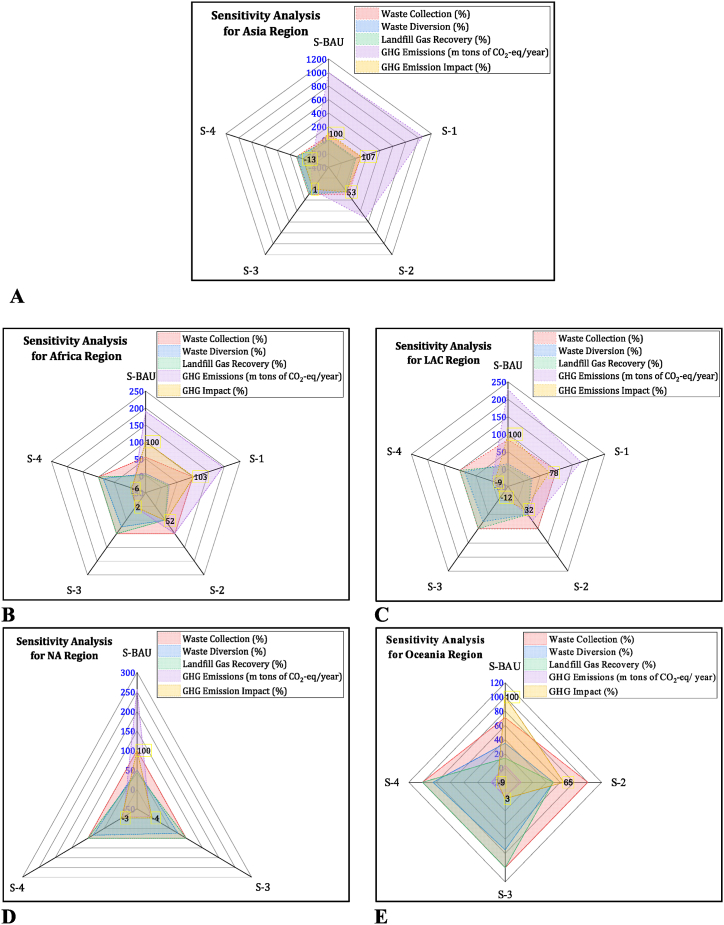
Regional sensitivity analysis for various scenarios. (A) Analysis for Asia (B) Analysis for Africa (C) Analysis for LAC. (D) Analysis for NA. (E) Analysis for Oceania region.

A region-wise sensitivity analysis is performed to determine the impacts of various scenarios on climate change under a given set of assumptions, such as waste collection efficiency, waste diversion percentages from landfilling, LFG recovery, and related GHG emissions. A 7 % and 3 % increase in GHG emissions are observed for Asia and African regions, respectively (Fig. 7A and B), under S-1 (25 % waste diversion) compared to S-BAU. This increase in GHG emissions is due to an increase in waste collection and related emissions from the transportation fleet to ensure 100 % collection efficiency and from waste disposal sites, which cater to 75 % of the collected waste with 20 % LFG recovery. However, the LAC region has an estimated 78 % GHG emissions impact, less than the S-BAU by 22 % in S-1 (Fig. 7C). Sensitivity analysis for S-2 explained the GHG emissions impact at 52 %, 53 %, 32 %, and 65 % for Africa, Asia, LCA, and Oceania regions, respectively, with waste diversion and LFG recovery rates at 50 %. These GHG emissions impact values are less than the S-BAU by 48 %, 47 %, 68 %, and 35 % in S-2 for Africa, Asia, LAC, and Oceania regions, as explained in Fig. 7A‒7E.

The GHG emissions impact is estimated at 2 %, 1 %, −12 %, −4%, and 3 % under S-3 at 75 % waste diversion with 100 % LFG recovery, and these impact values are less than the S-BAU by 98 %, 99 %, 112 %, 104 %, and 97 % for Africa, Asia, LAC, NA, and Oceania regions respectively as depicted in Fig. 7A‒7E. S-4, with 85 % waste diversion and 100 % LFG recovery, has GHG emissions impact value at −6%, −13 %, −13 %, −3%, and −9% which are less than the S-BAU by 106 %, 113 %, 113 %, 103 %, and 109 % for Africa, Asia, LAC, NA, and Oceania regions respectively as explained in Fig. 7A–E.

### 3.4 Waste treatment technologies and related climate impacts

**Fig. 8:**
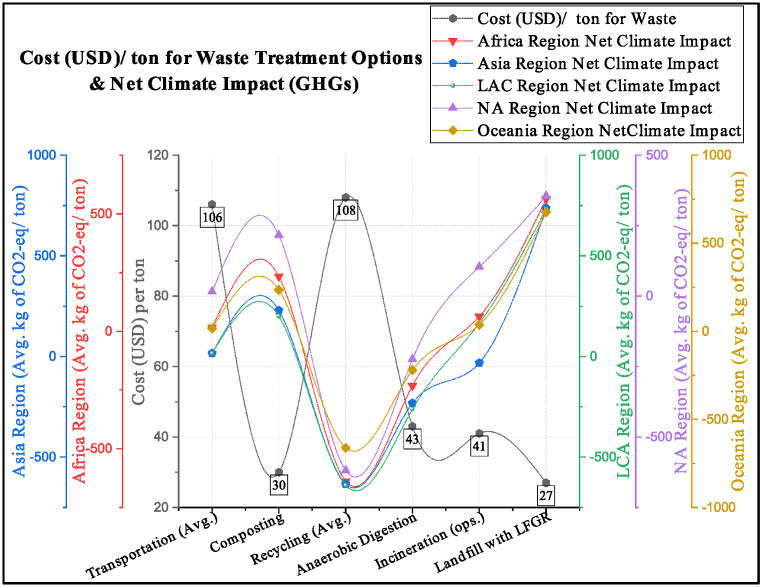
Economic analysis of various waste treatments and related climate impacts.

The economic analysis is performed for waste collection (USD 106 per ton) [50] and related treatment technologies, i.e., composting (USD 30 per ton) [51], recycling (USD 108 per ton) [52], anaerobic digestion (USD 43 per ton) [53], incineration (USD 41 per ton) [54] and landfilling with gas recovery (USD 27 per ton) [55]. It is found that the impact of GHG emissions is directly proportional to the operational cost per ton of the related waste treatment technology, such as recycling, which has the lowest impact on GHG emissions. However, it has the most expensive operations. Landfills with gas recovery have the lowest per-ton operational cost but significantly impact GHG emissions. A comparison of various waste treatment operating costs per ton with the impact of related GHG emissions is explained in Fig. 8.

### 3.5 Waste sector impacts on global climate change

Global temperature is responsible for fluctuations in the seal level, as evident from the last ice age when the atmospheric temperature was 5 °C colder than the current temperature with low sea level, i.e., 100 m below its current level [56,57]. Earth's temperature was reported to be 1°‒2 °C high, along with a 6 m rise in sea level today during the last interglacial period [57,58]. There is a possibility of raising the sea level by 3 m due to the collapse of West Antarctica's marine ice sheet over centuries due to oceanic and atmospheric warming [59]. Natural and anthropogenic activities are responsible for global warming and the rise in sea level as predicted by the latest approaches, i.e., semi-empirical cum satellite imagery, including observation data of Earth's surface and atmosphere shows that 0.8 °C rise in temperature during the twentieth century has trigger the rate of sea level rise as three times. Future warming scenarios predicted an increase in 1.4°–5.8 °C temperature between 1990 and 2100 that will rise 0.5–1.4 m sea level [38,39].

**Fig. 9:**
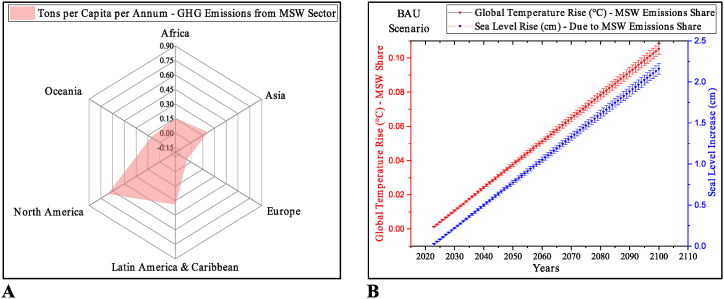
Waste sector and climatic impact. (A) Per capita GHG emissions from the waste sector. (B) The correlation between temperature and related seal level rise from MSW sector's related emissions under S-BAU.

The global contribution of the GHG emissions from the MSW sector (WSE_%share(GHG)_) is calculated as 3.42 % in the S-BAU (Equation (1)). Yearly GHG emissions from MSW sector in tons per capita in perspective of Earth's regions is depicted in Fig. 9A. The waste sector alone may contribute about 0.11 °C in GTR (AWSS_GTR(°C)_) at the end of the current century with a related 2.16 cm in SLR (AWSS_SLR(cm)_) as calculated by Equation (2‒6), and it may contribute in a loss to the coastal and marine ecosystem. The correlation between temperature rise and related sea level rise in perspective of GHG emissions from the MSW sector from 2024 to 2100 is depicted in Fig. 9B.

### 3.6 Waste sector ecological impact value

**Fig. 10:**
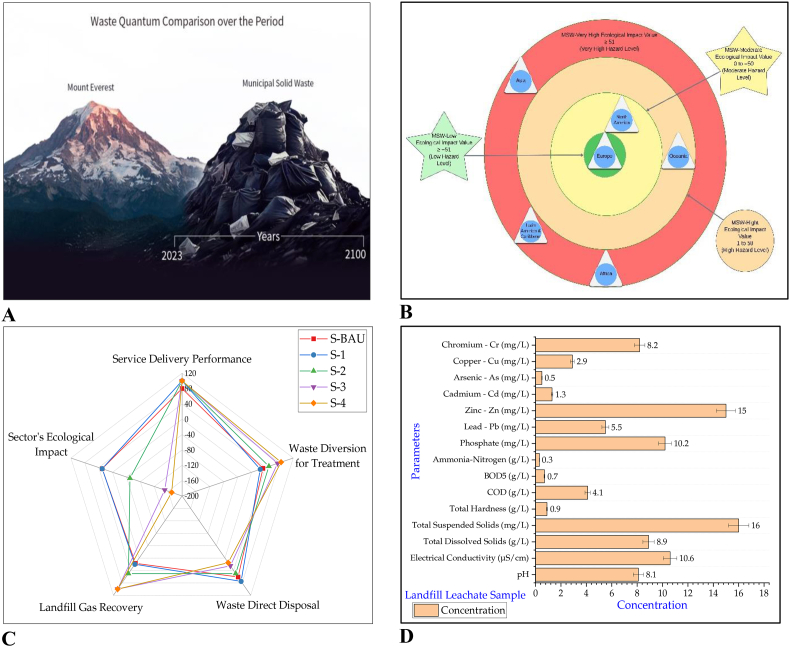
MSW-related ecotoxicity: (A) Graphical presentation of global waste generation over the period under business as usual. (B) The waste sector ecological impact value under business as usual. (C) The waste sector ecological impact value for each scenario. (D) Chemical composition of leachate at Sahiwal disposal facility.

Anthropogenic activities, i.e., industrialization cum urbanization, tend towards economic growth and are also responsible for impacting the health of the environment and ecosystem. These human-induced activities are considered accountable for disturbing the Earth's natural equilibrium of biodiversity and ecosystem, as evidenced by the study that has found a clue for climatic-induced polar motion [60]. MSW generation, collection and final disposal are regular assignments by the municipalities in the form of recurrent phenomena that challenge the natural local habitat. The cumulative quantity of MSW under S-BAU is projected to be 176 gigatons by 2100, almost equal to Mount Everest's weight [61]. However, disposal sites are unevenly distributed, and the graphical presentation of the global MSW quantity over the period is depicted in Fig. 10A.

MSW management is a critical concern in developing economies as service delivery level (SPD_%(sys)_) is found <80 % in regions such as Africa (57 %), Oceania (71 %), and Asia (72 %) (Equation (7)). Therefore, the uncollected waste (WCG_%(sys)_) in the form of gaps in service provision due to the non-availability of adequate funds in low‒income municipalities around the world is responsible for degrading the health of the ecosystem (Equation (8)). In most of the regions, the waste diversion for treatment (WD_%(ttt)_) is reported to be <50 %, and areas include Africa at 6 %, LAC at 15.5 %, Asia at 17.3 %, and Oceania at 35 % (Equation (9)). This low level of waste diversion from landfilling is mainly due to these countries' economic conditions, and international lenders need to invest in the waste sector to make it environmentally sustainable to achieve the carbon neutrality target.

The most prominent method of waste's final disposal is open dumping and landfilling (WDD_%(lfs)_), which is reported to be >50 % (Equation (10)) in most of Earth regions, i.e., Africa, Asia, LAC and Oceania. Open disposal and landfilling method is an economically cost-effective solution for low-income countries but a hazard to the environment and climate change in perspective of GHG emissions, as evident that dumpsites emissions contribution is 6–50 % in city-level total emissions [14]. LFG recovery (LFGR%) is also reported to be very low in developing economies, and it is <10 % (Equation (11) in Africa, Asia and LAC. The WS-EIV, such as WS%_(E±)_, is also calculated regarding the waste collection gap in services, unauthorized dumping, and open disposal without LFG recovery that contributes to the degradation of ecosystem health. The WS-EIV is further categorized as low (≥−51), moderate (0 to −50), high (1–50) and very high (≥51). Hotspot areas in perspective of WS-EIV are identified in Africa at the highest with 114 %, LAC at 76 %, Asia at 72 %, and Oceania at 45 % (high), as per Equation (12).

However, Europe and NA regions on Earth have attained sustainability in the waste sector with low and moderate WS-EIV under S-BAU (Fig. 10B), and other regions have great potential in achieving the targets to improve the health of the local ecosystem with low WS-EIV by implementing S-3 and, S-4 as depicted in Fig. 10C (see supplementary material). Organic waste is a more prominent component of MSW, which is reported to be >50 % in Africa, LAC, and Oceania, with about 47.7 % in Asia. The decomposition of organic waste produces LFG, potentially impacting leachate composition in disposal sites. The MSW management practices, including landfilling, need to be improved in low-income countries located in the tropical climatic regions of the world. Waste disposal facilities are a source of soil contamination, and leachate seepage into groundwater and the nearest water body due to the unavailability of geo-membrane and geotextile lining is responsible for the toxicity of the aquatic ecosystem [62].

Landfills and dumpsites in developing economies are a significant source for the accumulation and concentration of total dissolved solids (TDS), total suspended solids (TSS), dissolved oxygen (DO), inorganic salts, metals, ammonia and other toxicants. The presence of household electronic waste is a source of lead (Pb), cadmium (Cd), mercury (Hg), arsenic (As), copper (Cu), and zinc (Zn) that are not degraded in disposal sites and enter into environment and aquatic ecosystem [63].

A typical composition of leachate is presented in Fig. 10D, based on a sample collected from the disposal facility of Sahiwal, an intermediate populous city in central Punjab, Pakistan. The Leachate sample gives a pH value of 8.1 (Fig. 10D), which shows the old and resistant nature of leachate, i.e., methanogenic leachate and represents the conversion of organic acid into the gaseous phase [63]. Alkaline pH may also indicate a low concentration of free volatile acid and an indication of the old life of the disposal facility, i.e., >10 years. However, pH 6.5–8 is considered more suitable for aquatic animals. Leachate is responsible for altering the aquatic pH if it enters into nearby water bodies, as in the case of developing economies where disposal sites are mainly located near oceans, rivers, and canals, thus affecting the growth of aquatic biota [22].

Variations in the concentration of TDS directly impact the health of local biota. Physical variation in the appearance of TSS depends on the composition of plastic, paper, and kitchen waste at the disposal facility, which also impacts the penetration of light and is responsible for altering the temperature [22] and related gas emissions. A methanogenic phase of the disposal facility is also responsible for decreasing the concentration of BOD and COD of leachate. The concentration of COD at 4150 mg/L, NH_3_–N at 292 mg/L, PO_4_^−3^ at 10.2 mg/L, and biodegradability at medium-low (Fig. 10D) are an indication of methanation stage, and these values further indicate its entry towards next phase of neutralization [64]. Heavy metals concentration (mg/L) in leachate is indicated by the following order Zn > Cr > Pb > Cu > Cd > As (Fig. 10D), and are a significant toxicity concern for local ecosystem health.

The concentration of heavy metals, their route to exposure and receptor sensitivity are indicators responsible for impacting biodiversity health, i.e., acute to chronic [13]. This situation is further worsened as these trace elements tend to be toxic, bio-accumulated and have low degradability by exposure to the local ecosystem [65] and subsurface aquifers due to the formation of leachate plume in the absence of engineering sanitary landfills in developing economies. A matrix is presented in Table 3 to assess the sector impact value.

**Table 3:** Matrix for assessing the sector ecological impact value under BAU conditions.

Waste Sector Ecological Impact Value (%)	Hazard Impact Level	Waste Sector Indicators		Environmental/Climate Impacts of Waste Sector	Global Perspective
		Parameters	Value (%)		
≥ −51	Low	Service delivery performance	≥90	Near to zero sectorial carbon footprint. High ecosystem health from the perspective of leachate management.	Europe region
		Waste diversion	≥50		
		Direct waste disposal	<30		
		Landfill gas recovery	>30		
0 to −50	Moderate	Service delivery performance	≥80 to 89	Low carbon footprint with a degraded quality of the local ecosystem from the perspective of the waste sector.	North America region
		Waste diversion	≥30 to 49		
		Direct waste disposal	≥30 to 49		
		Landfill gas recovery	≥20 to 29		
1 to 50	High	Service delivery performance	≥70 to 79	The waste sector is a major contributor to city-level emissions with high leachate runoff.	Oceania region
		Waste diversion	≥20 to 29		
		Direct waste disposal	≥50 to 64		
		Landfill gas recovery	≥10 to 19		
≥51	Very High	Service delivery performance	<70	Dumpsites are a global hotspot of GHG emissions with toxic impacts on local ecosystem health.	Africa, Asia, and LAC regions
		Waste diversion	<20		
		Direct waste disposal	>65		
		Landfill gas recovery	<10		

### 3.7 Climate resilience approach in waste sector

The growth of urban areas and population has created many difficulties, particularly in South Asian countries, i.e., Pakistan and Bangladesh, where managing waste is challenging despite their little contribution to global GHG emissions [66]. Political and economic instability has pushed many developing economies below the poverty threshold, such as Pakistan. Despite this, the government of Pakistan prioritizes the construction of structures like bridges and roads rather than investing in essential public services such as sanitation and waste management [67]. Improving sanitation conditions is difficult due to the absence of national and local regulations with low enforcement. Under the enclosure approach of adaptation projects [66], the private sector was engaged in the Pakistan's waste sector, as summarized in Table 4.

**Table 4:** Lesson learned from private sector participation in the waste sector and related environmental liability in Pakistan.

Waste Project – Climate Resilience Action	Dimension/Scale	Beneficiary	Government Assets transferred to Private Sector	Environmental Consequences
Lahore compost	Local level	Private sector - focus on earning carbon credits	40-acre land on lease and free of cost waste delivery.	Low-quality organic compost. Free-of-cost handling of rejected waste by the municipality. Environmental liability on the part of the government.
Outsourcing of the waste sector in Lahore	City/district/municipality level	Private sector - focus on tonnage collection	Municipality crew, fix assets such as workshops and machinery	Payment linked with tonnage collected discouraged recycling and waste treatment projects. Waste accumulation at disposal facilities contributes 13 % of its share in city-level emissions [14]. Environmental liability on the part of the government.
Outsourcing of the waste sector in Rawalpindi	City/district/municipality level	Private sector - focus on tonnage collection	Municipality crew, fix assets such as workshops and machinery	Payment linked with tonnage collected discouraged recycling and waste treatment projects. Accumulation of waste at the disposal facility is responsible for GHG emissions. Environmental liability on the part of the government.
Quetta compost	Local level	Private sector	Land and waste delivery	In legal dispute.
Lahore RDF	Local level	Private cement sector	Waste delivery at a low cost.	Free-of-cost handling of rejected waste by the municipality. Environmental liability on the part of the government.
Outsourcing of the waste sector in Karachi	City/district/municipality level	Private sector	Municipality crew, fix assets such as workshops and machinery	Payment linked with tonnage collected discouraged recycling and waste treatment projects. Accumulation of waste at the disposal facility is responsible for GHG emissions. Environmental liability on the part of the government.
Islamabad RDF	Local level	Private cement sector	Land and waste delivery	Free-of-cost handling of rejected waste by the capital development authority (CDA). Environmental liability on the part of CDA.
Multan RDF	Local level	Private cement sector	Land and waste delivery	Free-of-cost handling of rejected waste by the municipality. Environmental liability on the part of the government.

**Fig. 11:**
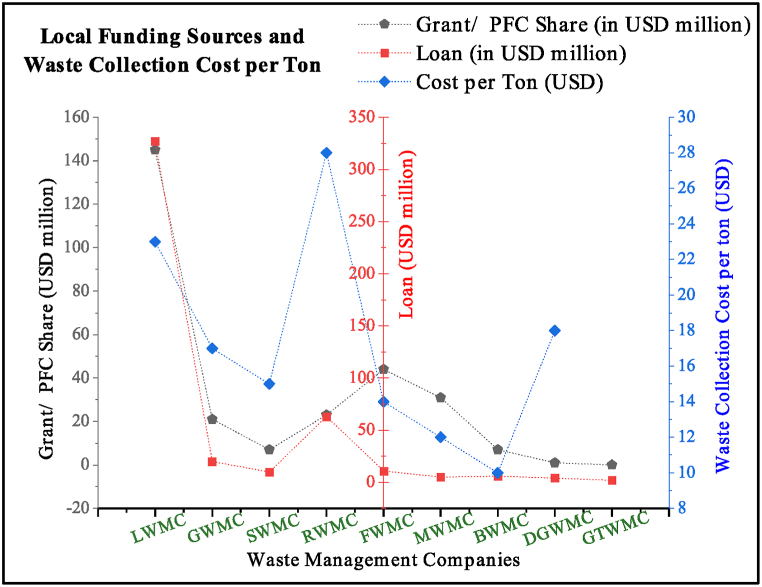
Funding sources, amount and tonnage collection cost of waste management companies.

However, these initiatives failed to sustain the waste industry in Pakistan because environmental liability was placed on the part of the government. In return, the private sector has earned profit from outsourced projects, captured the state land on lease, utilized the municipality crew, and earned carbon credits from compost manufactured without enhancing its quality, market, sector-related infrastructure development, and knowledge sharing to local governments. The state-run local waste sector has emerged as a major financial burden, with spending of USD 728 million, including 60 % loan money without any revenue (2011–2023), as explained in Fig. 11. The waste collection, haulage, and disposal cost per ton varies from USD 28 to USD 10 in Rawalpindi and Bahawalpur (Fig. 11). Open disposal of waste by all the municipalities is significant contributor of GHG in city level emissions and also responsible for degradation of land, surface, and groundwater quality.

South Asian communities can use indigenous knowledge to combat the negative impacts of the climate [68]. However, there is only a need for financial investment that can be recovered from the community against service provision as people are willing to pay more for better services. Segregation, recovery, and selling of plastic material from waste and an increase in tariff on imported products will be an additional source of revenue for the local government to invest in waste-related infrastructure development to sustain the sector [68]. Identified revenue streams will help the municipalities to adopt a climate resilience approach. The integration of cost-effective waste treatment technologies such as MRF for the recovery of recyclables and organic compost manufacturing will prove to be a step towards waste diversion in low-income countries to achieve the GHG emissions reduction targets.

**Fig. 12:**
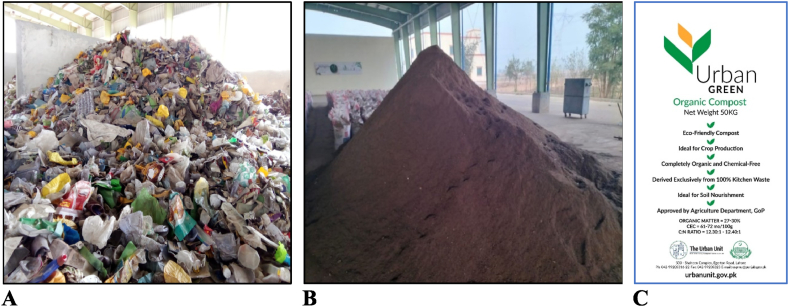
MRF in Sahiwal city. (A) Recovered recyclables from MSW. (B) Organic compost manufactured from MSW. (C) Compost brand for marketing in agriculture sector.

This locally manufactured MRF has the potential to sustain its operations in self-sufficient mode from the revenue money generated from the sale of recyclables (Fig. 12A) and compost (Fig. 12B). This kind of initiative is operational in the Sahiwal area, processing 40 t/d municipal waste in a single shift. This organic compost is notified as fit for its application in the agriculture sector to enhance the fertility of the soil (Fig. 12C). The organic matter, cation exchange capacity (CEC), and carbon to nitrogen ratio (C:N) are found at 30.1 %, 71.5 me/100g, and 12.4:1, respectively. The facility's net GHG emissions (saving) are calculated at −10,000 tons of CO_2_‒eq per annum and can contribute to NDCs. This type of initiative by the developing economies in the waste sector can help pay back to nature by reducing GHG emissions and improving soil texture and fertility for agricultural purposes.

This facility has much potential to replicate in other urban areas of developing economies. Thus, organic compost has the potential to contribute to reducing the climate vulnerability of poor populations of low-income countries by improving the soil fertility and related productivity of the agriculture sector [69], and there is a need to incorporate it into the national policies encouraging farmers to switch to organic fertilizers. The agriculture department has recommended 350 kg per acre of "urban green" organic compost to enhance soil fertility and crop growth.

Currently, the government of Pakistan has focused on transforming the urban areas into economic hubs with better civic services provision through governance and institutional reform programs with the support of international lenders such as the 10.13039/100004421World Bank and the 10.13039/100004425Asian Development Bank. Under these initiatives, waste collection equipment and machinery are being provided in 22 major cities of Punjab [70]. It is necessary to prioritize the establishment of facilities for treating municipal waste. The sustainability of the waste sector can only be achieved by the engagement of the private sector in waste treatment and landfilling, and financial investment in this infrastructure development and facilities establishments should be encouraged on built operate and transfer (BOT) mode. The gate/tipping fee may allow the private sector to recover the investment and operational costs to sustain the business.

**Fig. 13:**
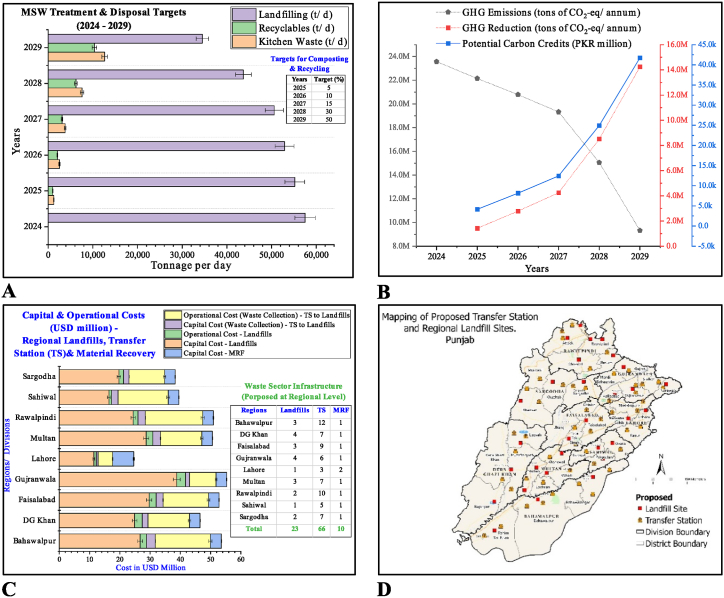
Proposed regional waste handling in Punjab. (A) Waste processing and recovery targets for treatment (B) Reduction in GHG emissions from waste diversion and potential carbon credits (C) Costing of landfills, transfer station and material recovery for the period of 10 years (D) Sites identified for establishment of transfer station cum MRF at district level in Punjab.

The MSW cycle includes waste collection, haulage, transportation, treatment, and disposal, with technological interventions to reduce GHG emissions and landfill gas recovery. This will support earning additional revenue from related carbon credits. Major cities in Pakistan, such as Lahore, Gujranwala, Rawalpindi, Faisalabad, Multan, Sialkot, and Bahawalpur, have great potential for the private sector to invest in GHG emissions reduction projects such as rehabilitation of old dumpsites for landfill methane recovery and the establishment of MRFs for composting and recovery of recyclables (Fig. 13A), which will pave the way towards climate funding with an opportunity to earn carbon credits as depicted in Fig. 13B.

Local municipal governments fail to provide services due to technical capacity and funding issues. Therefore, local policymakers have extended the scope of each district-level waste management company at the divisional level to improve waste collection efficiency, with liberty in decision-making at the company board level for outsourcing and investing in waste diversion. This step will help sustain the local waste sector. From the private sector participation perspective, the waste collection and disposal cost is proposed at USD 608 million per annum for the 36 Punjab districts. There are no single landfill sites in Pakistan except two cells in Lahore. Therefore, the establishment and operations of landfill sites are potential areas where the private sector can invest and recover the cost through tipping fees and the sale of landfill gas to some industries and related carbon credits from the perspective of GHG reduction. The capital and operational cost (10 years) of landfills, transfer station (TS) including waste collection from TS to landfills and establishing MRF are depicted in Fig. 13C. By considering the economy of waste business, various sites are identified for landfills development [71] based on regional/divisional concept as depicted in Fig. 13D. The transfer station cum MRF sites are also proposed in Punjab, keeping in view the distances from landfills to enhance the waste collection efficiency and recovery of recyclables and organic material in the Punjab province (Fig. 13D).

### 3.8 Nature-based solutions and adaptation in waste sector

Open waste disposal through landfilling is a more prominent method (70–90 %) in developing economies; however, closing and rehabilitating disposal facilities is a critical challenge and an important step to sustain the waste sector [72]. Open disposal sites are a continuous source of toxicity for the aquatic ecosystem as seepage and leachate runoff can pollute the nearby water body. The disposal sites adjacent to the water bodies are responsible for aquatic ecotoxicity. Most disposal facilities are located along water bodies or in river beds and are potential sources of soil, surface and underground contamination in Pakistan.

There is an urgent need to explore cost-effective solutions for rehabilitating existing and closed disposal facilities to improve ecosystem services, recreational opportunities for welfare of local people, enhancement in local property values, and environmental awareness at a global scale [73]. Therefore, phytocapping [74] is a cost-effective NbS to rehabilitate disposal sites for developing economies as a socially inclusive and good public acceptance alternative approach [75]. This method can stabilize the soil and remediate the landfill leachate [76]. The phytoremediation method [77], in combination with phytocapping, can work as hydrological control to infiltrate the rainwater to avoid erosion [78] and is a widely acceptable method to remediate landfills leachate and soil contamination against heavy metals [76]. This method has slow removal of contaminants. However, it can help establish a healthy ecosystem, improve air quality with a low lifecycle environmental footprint [75], and reduce surface methane emissions from disposal sites [79].

**Fig. 14:**
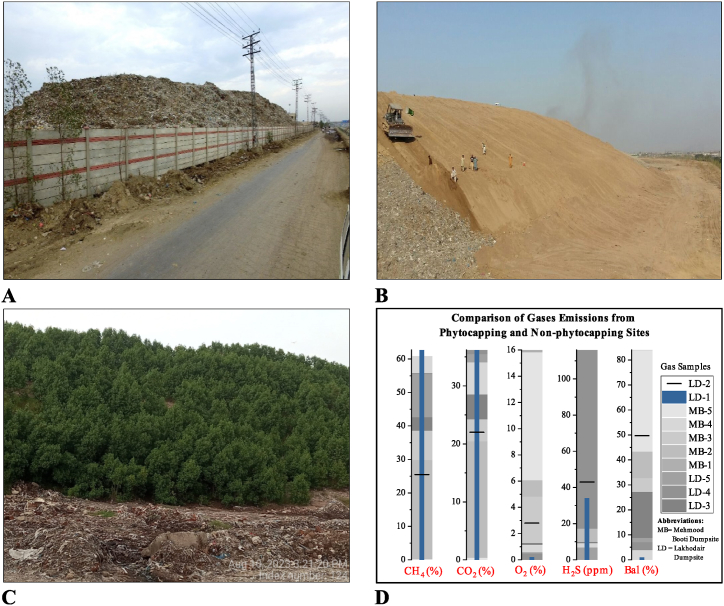
Phytoremediation initiative in Lahore city. (A) Mehmood Booti dumpsite before phytocapping. (B) Site preparation for rehabilitation. (C) Rehabilitation of old dumpsite with phytocapping technique. (D) Comparison of gas emission from disposal sites.

Lahore has rehabilitated the Mehmood Booti (MB) old disposal facility by adopting phytoremediation and phytocapping methods. The closed facility (Fig. 14A) is located in the northern part of the city and comprises 40 acres of land, with about 13 million tons of waste disposed of at the site from 1990 to 2016. About 20 % of the old site was covered with 2–4 feet of soil on top of the waste heap (Fig. 14B) and installed 14,500 Conocarpus (C.) erectus plants. The dumpsite is covered with *C*. erectus flourish well on the land (Fig. 14C). *C. erectus* is a well-tested plant that can remediate soil and leachate against multi-metal contamination such as Pb, Cr, Ni, and Cd [78]. *C. erectus* is a Lead hyper-accumulator and stabilizer for Cr, Ni, and Cd. This plant can accumulate higher Pb concentrations in shoots followed by roots; however, Cr, Ni, and Cd concentration is found more in roots than shoots [80].

The local community also appreciated the effort due to the aesthetics, regenerated green landscape with low capital expenditure (USD 0.2 million), helping city administration Lahore towards sustainability goals with land reclamation, and an improvement in the property value of the locality. The presence of gases at the MB site with phytocapping is determined with the help of a ""Geotech-5000 portable analyzer"" and compared with the gas composition of the Lakhodair (LD) disposal site without vegetated cover. Five samples are analyzed from each site, and results show that phytocapping technique can reduce the overall CH_4_, CO_2_ and H_2_S surface emissions by 46.7 %, 50.1 %, and 87.6 %, respectively, and the concentration of O_2_ is found more at 87.7 % as compared to a non-phytocapping site (Fig. 14D).

The NbS and adaptation will support reducing the city-level emissions from disposal facilities [14] with an opportunity to replicate the cost-effective approach to rehabilitating old disposal sites in urban areas of developing economies. Developed economies and regions have included relevant clauses in policy and legislation to achieve local waste treatment and recycling targets for waste diversion from landfilling. Therefore, developing economies must define goals and targets to divert the waste from open disposal towards waste treatment by incorporating legal infrastructure in prevailing legislations to support NbS for rehabilitating old dumpsites with carbon capture/financing opportunities [81]. International financial institutions (IFIs) can be crucial in addressing climate crises such as forest fires, drought, heatwaves, and flooding due to unusual global temperature rise in the last year [82]. As a mitigation strategy, regional policymakers can play their role in setting higher credits for carbon removal with NbS, and this approach must be included in the mitigation portfolio against climate crises from the perspective of low-income economies [83].

Developing economies such as Pakistan are more vulnerable to climatic changes, as evidenced by the flooding in 2010 and 2022 [84]. There is an urgent need for climate adaptation funds to sustain the social and economic condition of highly debited nations under the Copenhagen Accord and climate justice to support Pakistan with climate financing under article 9.2 of the Paris Accord that will help to justify the efforts with building confidence towards implementation of justice frame and criteria by the IFIs and lenders [85]. Innovative approaches can also be explored, such as utilizing carbon emissions reduction credits to pay off the national foreign debt, and this needs recognition by IFIs [86] that can play a significant role in motivating highly debited low‒income economies such as Pakistan, Ukraine, Egypt, Sri Lanka, El Salvador, Ghana, Lebanon, Tunisia, Maldives, and Zambia towards carbon neutrality, and poverty reduction with economic wellbeing.

This study will illuminate the path for policymakers and think tanks at the national and regional levels, showing how a phased transformation of the waste sector towards sustainability can yield significant benefits in the context of climate change. By adopting a targeted waste diversion approach, we can unlock the sector's potential to pay back nature' through cost-effective, value-added products that boost the productivity of the agriculture and industrial sectors. The adaptation of NbS to rehabilitate old dumpsites, particularly in developing economies, is emphasized to reduce GHG emissions and ground/surface water pollution. The modelling is performed based on waste data collected from current literature and waste diversion assumptions. However, further research is needed to verify and confirm the obtained results with actual data at national and regional levels.

## 4 Conclusion

Our research findings indicate that solid waste, in all its forms, has a significant toxic tendency to degrade the environment, with developing economies most at risk due to limited resources, technological barriers, poor infrastructure, and funding challenges. The decomposition of organic proportion of waste in disposal sites is a significant source of emitting GHG and leachate formation. Addressing waste sector problems and advocating for integrated sustainable waste management practices is imperative to protect biodiversity and preserve the intricate balance of ecosystems. Therefore, we have proposed scenarios with flexible timelines for implementing carbon neutrality with eco-friendly phytoremediation methods to rehabilitate old dumpsites from the perspective of low-income countries. The waste sector also has the potential to pay back to nature by improving soil fertility with organic compost, recovering recyclables, and reducing related emissions. However, developing economies can achieve the target of waste diversion from landfills by defining the short-, medium- and long‒term goals in legislation at the national and regional levels. By collaborating and joining forces, we may hope to reduce solid waste's unsettling impact on local ecology and pave the way towards a more environmentally friendly and thriving future for our planet and all its inhabitants.

## Funding

No internal and external funding was received for this study.

## Data availability

Relevant data is available in the supplementary material.

## CRediT authorship contribution statement

**Asif Iqbal:** Writing – original draft. **Abdullah Yasar:** Supervision. **Abdul-Sattar Nizami:** Supervision. **Imran Ali Sultan:** Visualization, Conceptualization. **Rafia Haider:** Project administration, Formal analysis. **Amtul Bari Tabinda:** Supervision. **Aman Anwer Kedwii:** Validation, Conceptualization. **Muhammad Murtaza Chaudhary:** Methodology. **Muhammad Usman Ghori:** Supervision, Software.

## Declaration of competing interest

The authors declare the following financial interests/personal relationships which may be considered as potential competing interests:

Asif Iqbal reports administrative support was provided by The Urban Unit, Lahore. Asif Iqbal reports a relationship with The Urban Unit, Lahore that includes: employment.
